# 3,4-Bis(4-meth­oxy­phen­yl)-2,5-dihydro-1*H*-pyrrole-2,5-dione

**DOI:** 10.1107/S1600536812014158

**Published:** 2012-04-06

**Authors:** Liangzhu Huang, Youqiang Li, Dongmei Gao, Zhenting Du

**Affiliations:** a College of Science, Northwest A&F University, Yangling, Shaanxi 712100, People’s Republic of China

## Abstract

In the title compound, C_18_H_15_NO_4_, the benzene rings form quite different dihedral angles [16.07 (1) and 59.50 (1)°] with the central pyrrole ring, indicating a twisted mol­ecule. Conjugation is indicated between the five- and six-membered rings by the lengths of the C—C bonds which link them [1.462 (3) and 1.477 (3) Å]. The most prominent feature of the crystal packing is the formation of inversion dimers *via* eight-membered {⋯HNCO}_2_ synthons.

## Related literature

For the use of 3,4-diaryl-substituted maleic imide derivatives as photochromic materials, see: Irie (2000 ▶); Liu *et al.* (2003 ▶). For the synthesis, see: Faul *et al.* (1999 ▶).
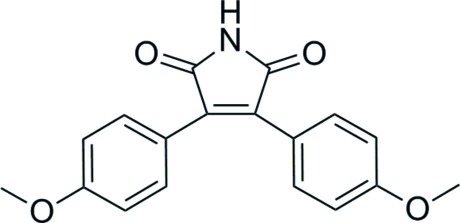


## Experimental

### 

#### Crystal data

C_18_H_15_NO_4_
*M* *_r_* = 309.31
Triclinic, 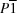

*a* = 6.030 (3) Å
*b* = 8.971 (5) Å
*c* = 14.023 (8) Å
α = 90.945 (6)°
β = 95.205 (5)°
γ = 97.862 (5)°
*V* = 748.0 (7) Å^3^
*Z* = 2
Mo *K*α radiation
μ = 0.10 mm^−1^
*T* = 296 K
0.69 × 0.23 × 0.19 mm

#### Data collection

Bruker APEXII CCD diffractometer
Absorption correction: multi-scan (*SADABS*; Bruker, 2009 ▶) *T*_min_ = 0.233, *T*_max_ = 0.982
3943 measured reflections
2607 independent reflections
1751 reflections with *I* > 2σ(*I*)
*R*_int_ = 0.034

#### Refinement

*R*[*F* ^2^ > 2σ(*F* ^2^)] = 0.051
*wR*(*F* ^2^) = 0.148
*S* = 1.03
2607 reflections
211 parameters
H-atom parameters constrained
Δρ_max_ = 0.16 e Å^−3^
Δρ_min_ = −0.18 e Å^−3^

### 

Data collection: *APEX2* (Bruker, 2009 ▶); cell refinement: *SAINT* (Bruker, 2009 ▶); data reduction: *SAINT*; program(s) used to solve structure: *SHELXS97* (Sheldrick, 2008 ▶); program(s) used to refine structure: *SHELXL97* (Sheldrick, 2008 ▶); molecular graphics: *SHELXTL* (Sheldrick, 2008 ▶); software used to prepare material for publication: *SHELXTL*.

## Supplementary Material

Crystal structure: contains datablock(s) I, global. DOI: 10.1107/S1600536812014158/tk5079sup1.cif

Structure factors: contains datablock(s) I. DOI: 10.1107/S1600536812014158/tk5079Isup2.hkl

Supplementary material file. DOI: 10.1107/S1600536812014158/tk5079Isup3.cdx

Supplementary material file. DOI: 10.1107/S1600536812014158/tk5079Isup4.cml

Additional supplementary materials:  crystallographic information; 3D view; checkCIF report

## Figures and Tables

**Table 1:** Hydrogen-bond geometry (Å, °)

*D*—H⋯*A*	*D*—H	H⋯*A*	*D*⋯*A*	*D*—H⋯*A*
N1—H1⋯O1^i^	0.86	2.03	2.882 (3)	168

Symmetry code: (i) 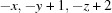.

**Fig. 1.**
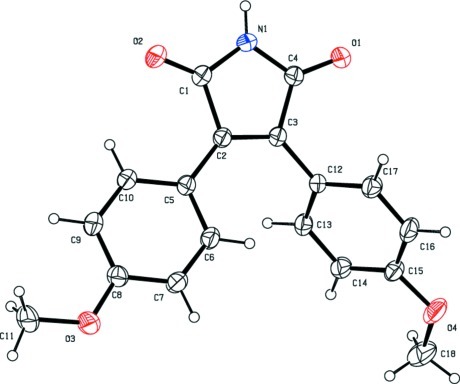
The molecular structure of (I). Displacement ellipsoids are drawn at the 30% probability level.

## supplementary crystallographic information

### Comment

3,4-Diaryl substituted maleic imide is a conjugated unit which has interesting optical and electronic properties. A number of 3,4-diaryl substituted maleic imide derivatives have been designed and synthesized to be used as photo-chromic materials (Irie, 2000; Liu *et al.*, 2003). In the course of exploring new photo-chromic compounds, we obtained an intermediate compound, 3,4-bis(4'-methoxyphenyl)maleic imide, (I). Herein, we report its structure.

The molecule was designed to feature two terminal methoxy group to enhance its solubility and to, later, enable fictionalization. The inter-planar angles between the two benzene rings connected with the maleic imide five-membered ring are different. The inter-planar angle between the benzene plane defined by C5–C10 and maleic imide plane is 16.07 (1) °. However, the inter-planar angle between the other benzene plane defined by C12–C17 and maleic imide plane is 59.50 (1) °. The lengths of the two single bonds connecting benzene groups and maleic imide are respectively 1.462 (3) Å (C2—C5) and 1.477 (3) Å (C3—C12), which are obviously shorter than typical C*sp*^3^—C*sp*^3^ single bond. This means that the bonding between the six-membered ring and the five-membered ring is quite conjugated.

### Experimental

For synthesis of imide (I), an improved procedure (Faul *et al.*, 1999) was followed using 4-methoxyphenylethylglyoxalate (2.1 g, 10 mmol), 4-methoxyphenylacetamide (1.65 g, 10 mmol) and freshly prepared NaOEt (40 mmol) in absolute ethanol (20 ml). The mixture was refluxed for 4 h and poured into diluted HCl. After conventional workup, purification was achieved by column chromatography (ethyl acetate/hexanes 1:1) to yield (I) (2.0 g, 65%) as a yellow solid. Crystals of (I) precipitated at 298 K from its methanol solution by slow evaporation.

### Refinement

All H atoms were placed in geometrically calculated positions and refined using a riding model with C—H = 0.93–0.96 Å and N—H = 0.86 Å and with *U*_iso_(H) = 1.2–1.5*U*_eq_(C, N).

### Crystal data

**Table:**

C_18_H_15_NO_4_	*Z* = 2
*M**_r_* = 309.31	*F*(000) = 324
Triclinic, *P*1	*D*_x_ = 1.373 Mg m^−^^3^
Hall symbol: -P 1	Melting point: 517 K
*a* = 6.030 (3) Å	Mo *K*α radiation, λ = 0.71073 Å
*b* = 8.971 (5) Å	Cell parameters from 1101 reflections
*c* = 14.023 (8) Å	θ = 2.7–23.6°
α = 90.945 (6)°	µ = 0.10 mm^−^^1^
β = 95.205 (5)°	*T* = 296 K
γ = 97.862 (5)°	Block, yellow
*V* = 748.0 (7) Å^3^	0.69 × 0.23 × 0.19 mm

### Data collection

**Table:**

Bruker APEXII CCD diffractometer	2607 independent reflections
Radiation source: fine-focus sealed tube	1751 reflections with *I* > 2σ(*I*)
Graphite monochromator	*R*_int_ = 0.034
φ and ω scans	θ_max_ = 25.2°, θ_min_ = 2.7°
Absorption correction: multi-scan (*SADABS*; Bruker, 2009)	*h* = −7→7
*T*_min_ = 0.233, *T*_max_ = 0.982	*k* = −10→8
3943 measured reflections	*l* = −16→16

### Refinement

**Table:**

Refinement on *F*^2^	Secondary atom site location: difference Fourier map
Least-squares matrix: full	Hydrogen site location: inferred from neighbouring sites
*R*[*F*^2^ > 2σ(*F*^2^)] = 0.051	H-atom parameters constrained
*wR*(*F*^2^) = 0.148	*w* = 1/[σ^2^(*F*_o_^2^) + (0.0769*P*)^2^] where *P* = (*F*_o_^2^ + 2*F*_c_^2^)/3
*S* = 1.03	(Δ/σ)_max_ < 0.001
2607 reflections	Δρ_max_ = 0.16 e Å^−^^3^
211 parameters	Δρ_min_ = −0.18 e Å^−^^3^
0 restraints	Extinction correction: *SHELXL97* (Sheldrick, 2008), Fc^*^=kFc[1+0.001xFc^2^λ^3^/sin(2θ)]^-1/4^
Primary atom site location: structure-invariant direct methods	Extinction coefficient: 0.114 (12)

### Special details

**Table:**

Geometry. All e.s.d.'s (except the e.s.d. in the dihedral angle between two l.s. planes) are estimated using the full covariance matrix. The cell e.s.d.'s are taken into account individually in the estimation of e.s.d.'s in distances, angles and torsion angles; correlations between e.s.d.'s in cell parameters are only used when they are defined by crystal symmetry. An approximate (isotropic) treatment of cell e.s.d.'s is used for estimating e.s.d.'s involving l.s. planes.
Refinement. Refinement of *F*^2^ against ALL reflections. The weighted *R*-factor *wR* and goodness of fit *S* are based on *F*^2^, conventional *R*-factors *R* are based on *F*, with *F* set to zero for negative *F*^2^. The threshold expression of *F*^2^ > σ(*F*^2^) is used only for calculating *R*-factors(gt) *etc*. and is not relevant to the choice of reflections for refinement. *R*-factors based on *F*^2^ are statistically about twice as large as those based on *F*, and *R*- factors based on ALL data will be even larger.

### Fractional atomic coordinates and isotropic or equivalent isotropic displacement parameters (Å^2^)

**Table:**

	*x*	*y*	*z*	*U*_iso_*/*U*_eq_	
C1	0.3834 (4)	0.3007 (3)	0.96603 (16)	0.0422 (6)	
C2	0.4781 (4)	0.3299 (2)	0.87029 (14)	0.0359 (6)	
C3	0.3710 (4)	0.4394 (2)	0.82907 (15)	0.0351 (5)	
C4	0.2055 (4)	0.4800 (3)	0.89376 (15)	0.0385 (6)	
C5	0.6460 (4)	0.2446 (2)	0.83558 (15)	0.0374 (6)	
C6	0.6830 (4)	0.2411 (3)	0.73879 (16)	0.0453 (6)	
H6	0.6022	0.2964	0.6961	0.054*	
C7	0.8349 (4)	0.1582 (3)	0.70524 (16)	0.0504 (7)	
H7	0.8558	0.1583	0.6403	0.060*	
C8	0.9589 (4)	0.0736 (2)	0.76676 (16)	0.0427 (6)	
C9	0.9229 (4)	0.0735 (3)	0.86255 (17)	0.0497 (7)	
H9	1.0021	0.0164	0.9047	0.060*	
C10	0.7698 (4)	0.1579 (3)	0.89605 (16)	0.0462 (6)	
H10	0.7486	0.1571	0.9609	0.055*	
C11	1.2389 (5)	−0.0910 (3)	0.7865 (2)	0.0646 (8)	
H11A	1.3177	−0.0288	0.8385	0.097*	
H11B	1.3454	−0.1313	0.7500	0.097*	
H11C	1.1421	−0.1721	0.8114	0.097*	
C12	0.4059 (4)	0.5231 (2)	0.74060 (14)	0.0347 (5)	
C13	0.6128 (4)	0.6031 (3)	0.72713 (16)	0.0449 (6)	
H13	0.7322	0.6006	0.7737	0.054*	
C14	0.6481 (4)	0.6866 (3)	0.64669 (16)	0.0465 (6)	
H14	0.7886	0.7405	0.6399	0.056*	
C15	0.4738 (4)	0.6893 (3)	0.57684 (16)	0.0441 (6)	
C16	0.2652 (4)	0.6106 (3)	0.58874 (17)	0.0537 (7)	
H16	0.1469	0.6125	0.5416	0.064*	
C17	0.2309 (4)	0.5294 (3)	0.66974 (16)	0.0458 (6)	
H17	0.0889	0.4782	0.6772	0.055*	
C18	0.6974 (5)	0.8516 (4)	0.4787 (2)	0.0779 (9)	
H18A	0.7448	0.9232	0.5307	0.117*	
H18B	0.6827	0.9037	0.4198	0.117*	
H18C	0.8070	0.7840	0.4750	0.117*	
N1	0.2275 (3)	0.3981 (2)	0.97460 (12)	0.0434 (5)	
H1	0.1543	0.4062	1.0239	0.052*	
O1	0.0729 (3)	0.57097 (18)	0.88000 (11)	0.0482 (5)	
O2	0.4269 (3)	0.2105 (2)	1.02547 (12)	0.0629 (6)	
O3	1.1072 (3)	−0.00307 (19)	0.72612 (12)	0.0586 (5)	
O4	0.4878 (3)	0.7688 (2)	0.49458 (12)	0.0668 (6)	

### Atomic displacement parameters (Å^2^)

**Table:**

	*U*^11^	*U*^22^	*U*^33^	*U*^12^	*U*^13^	*U*^23^
C1	0.0439 (14)	0.0460 (14)	0.0379 (13)	0.0089 (12)	0.0043 (11)	0.0091 (11)
C2	0.0362 (13)	0.0366 (12)	0.0347 (12)	0.0050 (10)	0.0019 (10)	0.0052 (10)
C3	0.0356 (13)	0.0361 (12)	0.0340 (12)	0.0072 (10)	0.0024 (10)	0.0059 (9)
C4	0.0360 (13)	0.0406 (13)	0.0399 (13)	0.0072 (11)	0.0052 (10)	0.0066 (10)
C5	0.0385 (13)	0.0367 (12)	0.0380 (12)	0.0077 (11)	0.0041 (10)	0.0076 (10)
C6	0.0568 (16)	0.0453 (14)	0.0373 (13)	0.0193 (12)	0.0045 (11)	0.0088 (11)
C7	0.0687 (18)	0.0513 (15)	0.0371 (13)	0.0240 (14)	0.0124 (12)	0.0089 (11)
C8	0.0464 (15)	0.0342 (12)	0.0499 (14)	0.0103 (11)	0.0101 (11)	0.0052 (11)
C9	0.0565 (17)	0.0496 (15)	0.0465 (14)	0.0182 (13)	0.0045 (12)	0.0147 (12)
C10	0.0524 (15)	0.0535 (15)	0.0373 (13)	0.0196 (13)	0.0084 (11)	0.0111 (11)
C11	0.071 (2)	0.0505 (16)	0.081 (2)	0.0338 (15)	0.0144 (16)	0.0137 (14)
C12	0.0351 (13)	0.0382 (13)	0.0331 (11)	0.0121 (10)	0.0041 (10)	0.0071 (9)
C13	0.0369 (14)	0.0571 (15)	0.0410 (13)	0.0097 (12)	−0.0013 (11)	0.0134 (11)
C14	0.0400 (14)	0.0529 (15)	0.0466 (14)	0.0039 (12)	0.0062 (11)	0.0142 (12)
C15	0.0542 (16)	0.0460 (14)	0.0346 (12)	0.0132 (12)	0.0062 (11)	0.0115 (10)
C16	0.0490 (16)	0.0707 (18)	0.0397 (14)	0.0086 (14)	−0.0083 (12)	0.0152 (12)
C17	0.0390 (14)	0.0530 (15)	0.0436 (14)	0.0015 (12)	−0.0001 (11)	0.0105 (11)
C18	0.090 (2)	0.079 (2)	0.0653 (19)	0.0022 (19)	0.0234 (17)	0.0333 (16)
N1	0.0472 (12)	0.0525 (12)	0.0352 (10)	0.0163 (10)	0.0135 (9)	0.0107 (9)
O1	0.0461 (10)	0.0555 (10)	0.0485 (10)	0.0206 (9)	0.0112 (8)	0.0133 (8)
O2	0.0737 (13)	0.0724 (13)	0.0524 (10)	0.0323 (11)	0.0196 (9)	0.0322 (10)
O3	0.0685 (12)	0.0565 (11)	0.0605 (11)	0.0324 (10)	0.0198 (9)	0.0118 (9)
O4	0.0743 (14)	0.0783 (13)	0.0479 (11)	0.0073 (11)	0.0062 (10)	0.0328 (10)

### Geometric parameters (Å, º)

**Table:**

C1—O2	1.208 (2)	C11—O3	1.429 (3)
C1—N1	1.380 (3)	C11—H11A	0.9600
C1—C2	1.519 (3)	C11—H11B	0.9600
C2—C3	1.357 (3)	C11—H11C	0.9600
C2—C5	1.462 (3)	C12—C13	1.382 (3)
C3—C12	1.477 (3)	C12—C17	1.390 (3)
C3—C4	1.485 (3)	C13—C14	1.381 (3)
C4—O1	1.224 (3)	C13—H13	0.9300
C4—N1	1.367 (3)	C14—C15	1.373 (3)
C5—C10	1.395 (3)	C14—H14	0.9300
C5—C6	1.396 (3)	C15—O4	1.369 (3)
C6—C7	1.366 (3)	C15—C16	1.381 (3)
C6—H6	0.9300	C16—C17	1.375 (3)
C7—C8	1.393 (3)	C16—H16	0.9300
C7—H7	0.9300	C17—H17	0.9300
C8—O3	1.358 (3)	C18—O4	1.413 (3)
C8—C9	1.380 (3)	C18—H18A	0.9600
C9—C10	1.380 (3)	C18—H18B	0.9600
C9—H9	0.9300	C18—H18C	0.9600
C10—H10	0.9300	N1—H1	0.8600
			
O2—C1—N1	124.0 (2)	O3—C11—H11C	109.5
O2—C1—C2	129.2 (2)	H11A—C11—H11C	109.5
N1—C1—C2	106.80 (19)	H11B—C11—H11C	109.5
C3—C2—C5	131.08 (19)	C13—C12—C17	117.5 (2)
C3—C2—C1	106.54 (19)	C13—C12—C3	120.88 (19)
C5—C2—C1	122.34 (19)	C17—C12—C3	121.5 (2)
C2—C3—C12	131.28 (19)	C14—C13—C12	122.1 (2)
C2—C3—C4	108.34 (18)	C14—C13—H13	118.9
C12—C3—C4	120.22 (18)	C12—C13—H13	118.9
O1—C4—N1	124.7 (2)	C15—C14—C13	119.4 (2)
O1—C4—C3	127.6 (2)	C15—C14—H14	120.3
N1—C4—C3	107.73 (19)	C13—C14—H14	120.3
C10—C5—C6	116.7 (2)	O4—C15—C14	124.7 (2)
C10—C5—C2	122.1 (2)	O4—C15—C16	115.7 (2)
C6—C5—C2	121.1 (2)	C14—C15—C16	119.6 (2)
C7—C6—C5	121.6 (2)	C17—C16—C15	120.6 (2)
C7—C6—H6	119.2	C17—C16—H16	119.7
C5—C6—H6	119.2	C15—C16—H16	119.7
C6—C7—C8	121.0 (2)	C16—C17—C12	120.8 (2)
C6—C7—H7	119.5	C16—C17—H17	119.6
C8—C7—H7	119.5	C12—C17—H17	119.6
O3—C8—C9	125.3 (2)	O4—C18—H18A	109.5
O3—C8—C7	116.1 (2)	O4—C18—H18B	109.5
C9—C8—C7	118.5 (2)	H18A—C18—H18B	109.5
C8—C9—C10	120.2 (2)	O4—C18—H18C	109.5
C8—C9—H9	119.9	H18A—C18—H18C	109.5
C10—C9—H9	119.9	H18B—C18—H18C	109.5
C9—C10—C5	122.0 (2)	C4—N1—C1	110.48 (18)
C9—C10—H10	119.0	C4—N1—H1	124.8
C5—C10—H10	119.0	C1—N1—H1	124.8
O3—C11—H11A	109.5	C8—O3—C11	118.18 (19)
O3—C11—H11B	109.5	C15—O4—C18	118.1 (2)
H11A—C11—H11B	109.5		
			
O2—C1—C2—C3	178.3 (2)	C6—C5—C10—C9	0.5 (3)
N1—C1—C2—C3	−0.8 (2)	C2—C5—C10—C9	177.8 (2)
O2—C1—C2—C5	0.4 (4)	C2—C3—C12—C13	−56.6 (3)
N1—C1—C2—C5	−178.69 (19)	C4—C3—C12—C13	118.2 (2)
C5—C2—C3—C12	−8.5 (4)	C2—C3—C12—C17	126.1 (3)
C1—C2—C3—C12	173.9 (2)	C4—C3—C12—C17	−59.2 (3)
C5—C2—C3—C4	176.3 (2)	C17—C12—C13—C14	−0.2 (3)
C1—C2—C3—C4	−1.4 (2)	C3—C12—C13—C14	−177.7 (2)
C2—C3—C4—O1	−177.7 (2)	C12—C13—C14—C15	−0.9 (4)
C12—C3—C4—O1	6.5 (3)	C13—C14—C15—O4	179.4 (2)
C2—C3—C4—N1	3.1 (2)	C13—C14—C15—C16	1.0 (4)
C12—C3—C4—N1	−172.74 (19)	O4—C15—C16—C17	−178.6 (2)
C3—C2—C5—C10	166.7 (2)	C14—C15—C16—C17	−0.1 (4)
C1—C2—C5—C10	−16.0 (3)	C15—C16—C17—C12	−1.0 (4)
C3—C2—C5—C6	−16.1 (4)	C13—C12—C17—C16	1.1 (4)
C1—C2—C5—C6	161.3 (2)	C3—C12—C17—C16	178.6 (2)
C10—C5—C6—C7	−0.7 (4)	O1—C4—N1—C1	177.1 (2)
C2—C5—C6—C7	−178.1 (2)	C3—C4—N1—C1	−3.6 (3)
C5—C6—C7—C8	0.1 (4)	O2—C1—N1—C4	−176.4 (2)
C6—C7—C8—O3	−179.2 (2)	C2—C1—N1—C4	2.8 (2)
C6—C7—C8—C9	0.9 (4)	C9—C8—O3—C11	−0.3 (3)
O3—C8—C9—C10	179.0 (2)	C7—C8—O3—C11	179.8 (2)
C7—C8—C9—C10	−1.1 (4)	C14—C15—O4—C18	1.5 (4)
C8—C9—C10—C5	0.4 (4)	C16—C15—O4—C18	179.9 (2)

### Hydrogen-bond geometry (Å, º)

**Table:**

*D*—H···*A*	*D*—H	H···*A*	*D*···*A*	*D*—H···*A*
N1—H1···O1^i^	0.86	2.03	2.882 (3)	168

Symmetry code: (i) −*x*, −*y*+1, −*z*+2.
